# Pyro‐Phototronic Effect Induced Circularly Polarized Light Detection with a Broadband Response

**DOI:** 10.1002/advs.202404403

**Published:** 2024-07-23

**Authors:** Qianwen Guan, Zeng‐Kui Zhu, Huang Ye, Chengshu Zhang, Hang Li, Chengmin Ji, Xitao Liu, Junhua Luo

**Affiliations:** ^1^ State Key Laboratory of Structure Chemistry Fujian Institute of Research on the Structure of Matter Chinese Academy of Sciences Fuzhou Fujian 350002 P. R. China; ^2^ Fujian Science and Technology Innovation Laboratory for Optoelectronic Information of China, Fuzhou Fujian 350108 China; ^3^ University of Chinese Academy of Sciences Beijing 100049 P. R. China

**Keywords:** broadband response, chiral polar hybrid perovskites, circularly polarized light detection, pyro‐phototronic effect

## Abstract

Photopyroelectric‐based circularly polarized light (CPL) detection, coupling the pyro‐phototronic effect and chiroptical phenomena, has provided a promising platform for high‐performance CPL detectors. However, as a novel detection strategy, photopyroelectric‐based CPL detection is currently restricted by the short‐wave optical response, underscoring the urgent need to extend its response range. Herein, visible‐to‐near‐infrared CPL detection induced by the pyro‐phototronic effect is first realized in chiral‐polar perovskites. Specifically, chiral‐polar multilayered perovskites (S‐BPEA)_2_FAPb_2_I_7_ (**1‐S**, S‐BPEA = (*S*)‐1‐4‐Bromophenylethylammonium, FA = formamidinium) with spontaneous polarization shows intrinsic pyroelectric and photopyroelectric performance. Strikingly, combining its merits of the pyro‐phototronic effect and intrinsic wide‐spectrum spin‐selective effect, chiral multilayered **1‐S** presents efficient photopyroelectric‐based broadband CPL detection performance spanning 405–785 nm. This research first realizes photopyroelectric‐based infrared CPL detection and also sheds light on developing high‐performance broadband CPL detectors based on the pyro‐phototronic effect in the fields of optics, optoelectronics, and spintronics.

## Graphical abstract

A 2D multilayered formamidine‐based chiral halide perovskites (*S*‐BPEA)_2_FAPb_2_I_15_ (S‐BPEA = (*S*)−1‐4‐Bromophenylethylamium, FA = formamidnium), combining the intrinsic merits of the chiroptical activity and pyro‐phototronic effect, realized the broadband pyroelectric‐based circularly polarized light (CPL) detection spinning visible to near‐infrared (NIR) range. The work first identified the potential of the pyro‐phototronic effect in chiral polar multilayered perovskites for efficient broadband CPL detection.

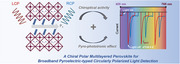

## 1 Introduction

Circularly polarized light (CPL) sensitive photodetectors, characterized by differentiating the left‐handed and right‐handed CPL light, have shown extensive applications in optical communication, drug screening, and security surveillance.^[^ ^1^ ^]^ However, traditional CPL detectors based on achiral inorganic semiconductors inevitably require installing optical components to distinguish CPL, which increases the cost and difficulty of integration and miniaturization.^[^ ^2^ ^]^ Therefore, exploiting new material systems with intrinsic chirality is significant to developing CPL‐sensitive photodetectors. Recently, two‐dimensional chiral organic‐inorganic hybrid perovskites (2D chiral OIHPs), have demonstrated remarkable potential in direct CPL detection owing to their inherent chiral structure, handedness‐sensitive optical absorption, and efficient spin carrier transport ability.^[^ ^3^ ^]^ To exploit high‐performance 2D chiral OIHPs‐based CPL detectors, significant efforts are made, such as designing mixed‐metal chiral perovskites^[^ ^4^ ^]^ or halogen‐substituted chiral cation‐constructed chiral perovskites^[^ ^5^ ^]^ and designing heterostructure,^[^ ^6^ ^]^ nanowire arrays,^[^ ^7^ ^]^ et.al. These methods are prone to effectively modify the selective CPL absorption and spin carrier transport of CPL detectors. However, current CPL detectors still require further development in pursuit of high performance, simple fabrication, and low cost.

Recently, the pyro‐phototronic effect has been exploited in 2D chiral OIHPs, which is a new emerging technology and promising for efficient CPL detection.^[^ ^8^ ^]^ Specifically, the pyro‐phototronic effect, a three‐way coupling between the semiconductor, photoexcitation, and pyroelectricity, can manipulate the processes of light‐induced carriers to achieve higher energy conversion and improve CPL detection performance.^[^ ^9^ ^]^ For instance, Li et.al reported a pair of 2D chiral‐polar perovskites S/R‐[(4‐aminophenyl)ethylamine]_2_AgBiI_8_·0.5H_2_O, showing a unique photopyroelectric‐based CPL detection performance with an impressive *g*_Iph_ of 0.27.^[^ ^8^ ^]^ However, limited by the optical bandgap of 2D monolayer chiral OIHPs, reported photopyroelectric‐based CPL detectors mainly focus on the UV–vis region, whereas near‐infrared (NIR) CPL detection remains a major challenge for chiral hybrid perovskite systems.^[^ ^10^ ^]^ Fortunately, beneficial from their structure tunability, 2D multilayered chiral OHIPs with a narrow bandgap and wide spin‐selective absorption range can be constructed by inserting small cations into the inorganic framework, such as formamidine (FA^+^), cesium (Cs^+^), ethylamine (EA^+^), and methylamine (MA^+^) et al.^[^ ^11^ ^]^ More importantly, combining their pyro‐phototronic effect and wideband chiroptical activity, 2D multilayered chiral polar OIHPs show tremendous potential for efficient broadband photoelectric‐based CPL detection.

Herein, the pyro‐phototronic effect‐induced broadband CPL detection from the visible to the near‐infrared region is first realized in 2D chiral halide perovskites. Specifically, the formamidine‐based multilayered (S‐BPEA)_2_FAPb_2_I_7_ (**1‐S** S‐BPEA = (*S*)−1‐4‐Bromophenylethylammonium, FA = formamidinium) with natural chiral polar structure shows the intrinsic pyroelectric properties and exceptional photopyroelectric response from 405 to 1550 nm. Importantly, combining the merits of wide chiroptical activity up to NIR and intrinsic pyro‐phototronic effects, **1‐S** displays notable broadband photopyroelectric‐based CPL detection at 405, 520, 637, and 785 nm, with the anisotropic factor of 0.24, 0.29, 0.26, and 0.20 respectively. Based on the intrinsic spin‐selective effect of **1‐S** for NIR light, this work first realizes NIR photopyroelectric‐based CPL detection, providing a new strategy for broadband high‐performance CPL detection.

## 2 Results and Discussion

### 2.1 Basic Crystal Characterization

**Figure 1:**
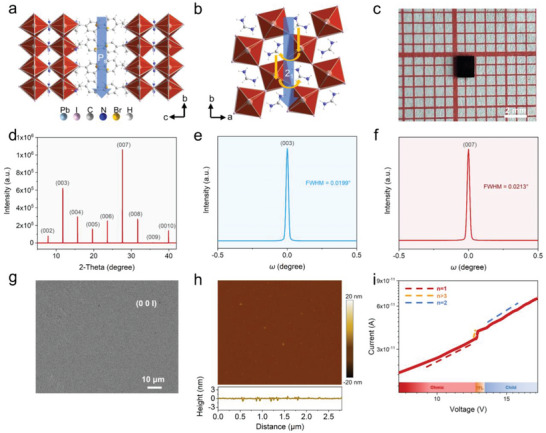
The primary characterization of 2D multilayered chiral polar perovskite (*S*‐BPEA)_2_FAPb_2_I_7_ (**1‐S**). a) The crystal structure of **1‐S** (the blue arrow refers to the polarization direction). b) The chiral polar structure with intrinsic 2_1_ axes in **1‐S**. c) The black‐red single crystal of **1‐S**. d) The XRD spectra on the top face of the single crystal of **1‐S**. e) High‐resolution XRD rocking curves for the diffraction peaks at (003) and f) (007), respectively. g) The SEM spectra of **1‐S**. h) The AFM spectra of **1‐S**. i) The trap density of **1‐S**.

Compared with single‐layered counterparts, chiral polar multilayered 2D OIHPs with natural polar structure, and wider chiroptical activity, are potential broadband photopyroelectric‐typed CPL detection materials.^[^ ^12^ ^]^ Among them, the rare formamidine‐based multilayered OIHPs have shown the outstanding advantages of better carrier transition and narrower bandgap, which is mainly attributed to the suitable size of formamidinium (FA^+^) to balance the distortion of the inorganic skeleton, the strong hydrogen bonding interactions between FA^+^ and inorganic framework, and the possible participation of FA^+^ as a conjugated cation to form band edges^[^ ^13^ ^]^ Thus, the formamidine‐based chiral multilayered (S‐BPEA)_2_FAPb_2_I_7_ (**1‐S**) (S‐BPEA = (*S*)−1‐4‐Bromophenylethylammonium), with intrinsic polar structure and narrow bandgap, was selected to exploit its potential for broadband photopyroelectric‐based CPL detection. By reacting the stoichiometric amounts of S‐BPEA, FA, and Pb(Ac)_2_ ·3H_2_O in hydriodic acid (HI), the reported black‐red single crystal **1‐S** was successfully synthesized by a simple cooling program (details are shown in the Supporting Information). The powder X‐ray diffraction (PXRD) spectra verified its purity and phase stability after 90 days, in which the experimental pattern matches well with the simulated one (Figure S1a, Supporting Information).^[^ ^14^ ^]^ The thermal gravity measurement identified its excellent thermal stability with a high decomposition temperature of 503 K (Figure S1b, Supporting Information). As revealed by the single‐crystal XRD analysis, **1‐S** crystallizes in the chiral polar space group (*P*2_1_) (Table S1, Supporting Information). The intrinsic polar structure endows **1‐S** a pyroelectric effect, which can enable efficient conversion of temperature fluctuations into electric charges, and lays a foundation for **1‐S** with a pyro‐phototronic effect to break the bandgap limitation for broadband photopyroelectric detection (**Figure** **1**a; Figure S2, Supporting Information).^[^ ^15^ ^]^ Moreover, the intrinsic *P*2_1_ chiral spiral structure along the *b*‐axis can further confer **1‐S** intrinsic chiroptical activity, which will combine the merits of the pyro‐phototronic effect for CPL‐sensitive photopyroelectric detection (Figure 1b; Figure S3, Supporting Information).^[^ ^8^, ^16^ ^]^ Therefore, based on these experimental conceptions, the single crystal of **1‐S** with dimensions of 2 × 2 × 0.5 mm^3^ was synthesized (Figure 1c). Bulk XRD scans display strong and sharp (00l) peaks, indicating high‐crystal quality and well‐oriented growth (Figure 1d). The narrow full width at half maximums (FWHM) of (003) and (007) peaks were further analyzed by the high‐resolution X‐ray rocking‐curve presents (0.0199° and 0.0213°, respectively) (Figure 1e,f), which is smaller than those of reported high‐quality single‐crystal photoelectric detectors, including (BDA)PbI_4_^[^ ^17^ ^]^ (0.089° in (00l) plane), MA_3_Bi_2_I_9_ (0.026° in (002) plane)^[^ ^18^ ^]^ and Cs_2_AgBiBr_6_ single crystal^[^ ^19^ ^]^ (0.0745° in (111) plane). The flat and smooth (00l) plane are shown in the scan electronic microscope (SEM) image and the atom force microscope (AFM) image, which also demonstrates the good crystal quality of **1‐S** (Figure 1g,h).^[^ ^20^ ^]^ Then, adopting the space charge limited current (SCLC) method, the trap density (*n*_trap_) of **1‐S** single crystal was quantitatively calculated.^[^ ^21^ ^]^ As shown in Figure 1i, the Ohmic area, the trap filling limit (TFL) area, and the Child's area, are shown in the logarithmic current‐voltage curve. In the TFL region, the current exhibits a rapid nonlinear increase with increasing voltage (I ∝ V^n^, n > 3). The trap density (*n*_trap_) is calculated by the equation *n*_trap_ = 2εε_0_V_TFL_/eL^2^, where ε is the relative dielectric constant, ε_0_ is the vacuum dielectric constant, e is the elemental charge, and L is the distance between two conductive electrodes. The *n*_trap_ of **1‐S** is estimated to be 3.5 × 10^10^ cm^−3^, smaller than some traditional inorganic photodetection materials, including Si (≈10^14^ cm^−3^),^[^ ^21^ ^]^ and CdTe/CdS (≈10^13^ cm^−3^)^[^ ^22^ ^]^ and comparable with high‐quality 3D MAPbX_3_ (X = Cl, Br, I; ≈10^10^ cm^−3^).^[^ ^23^ ^]^ Consequently, high‐quality single‐crystal **1‐S** shows a flat surface and lower trap density, which is desirable for efficient carrier transition and high‐performance CPL detection.

### 2.2 Polar Structure‐Induced Related Properties

**Figure 2:**
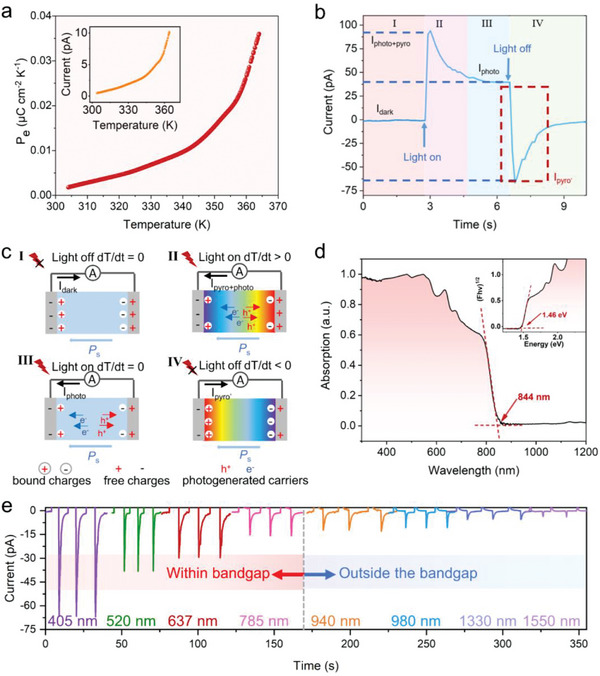
The polar structure induced related properties of **1‐S**. (a) The pyroelectric measurement of **1‐S** along the polar axis. b) The photopyroelectric spectra of **1‐S** along the *b*‐axis under 405 nm. c) The mechanism of photopyroelectric effect. d) The absorption and bandgap of **1‐S** e) The broadband photoexcited pyroelectric spectra of **1‐S** from 405 to 1550 nm with the light power of 184 mW cm^−2^.

Of the 21 non‐centrosymmetric point groups, 20 exhibit direct piezoelectricity (except for the cubic point group 432), and 10 are polar point groups that exhibit both piezoelectricity and pyroelectricity^[^ ^24^ ^]^ (Figure S2, Supporting Information). Thus, based on the polar structure of high‐quality single crystals **1‐S**, we explored its relevant properties, including piezoelectric, pyroelectric, and photopyroelectric. As shown in Figure S4 (Supporting Information), **1‐S** shows a significant piezoelectric response along the polar axis with the direct piezoelectric coefficient (*d*_33_) value of 6.1 pC N^−1^, demonstrating its non‐centrosymmetric crystal structure that can generate charges in response to mechanical forces.^[^ ^24^ ^]^ Meanwhile, **1‐S** displays remarkable variable‐temperature pyroelectric behavior along the polar axis, in which the current is enhanced with the increased temperature under zero bias (**Figure** **2**a). Based on the current‐temperature curve, the pyroelectric coefficient (*P*_e_), as an important parameter, is estimated (Calculation details are shown in the SI). At room temperature, **1‐S** has a *P*_e_ value of 1.8 × 10^−3^ µC cm^−2^ K^−1^, which is comparable to that of the conventional pyroelectric material PVDF (≈2.7 × 10^−3^ µC cm^−2^ K^−1^)^[^ ^25^ ^]^ and reported 2D OIHPs efficient pyroelectric material including (bromobenzylammonium)_2_(EA)_2_Pb_3_Br_10_ (4.0 × 10^−3^ µC cm^−2^ K^−1^),^[^ ^26^ ^]^ S/R‐[(4‐aminophenyl)ethylamine]_2_AgBiI_8_·0.5H_2_O (≈10^−3^ µC cm^−2^ K^−1^)^[^ ^8^ ^]^ and (isoamylammonium)_2_EA_2_Pb_3_Cl_10_ (1.0 × 10^−3^ µC cm^−2^ K^−1^),^[^ ^27^ ^]^ showing an intriguing potential for temperature‐sensitive photopyroelectric detection.

### 2.3 Photopyroelectric and Photopyroelectric‐Based CPL Detection

**Figure 3:**
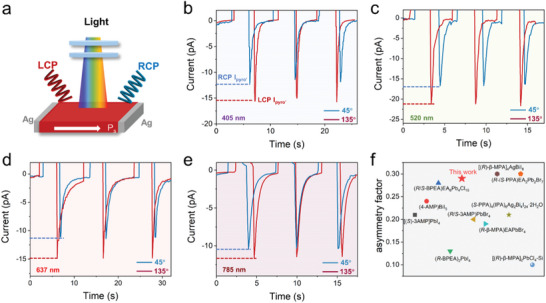
The photopyroelectric‐based self‐driven CPL detection. a) Schematic diagram of photopyroelectric‐based CPL detection b) The broadband pyro‐phototronic effect induced self‐driven CPL detection under 405 nm, c) 520 nm, d) 637 nm, e) 785 nm with the light power of 65.5, 92.3, 85.2, and 120 mW cm^−^^2^, respectively. f) The comparison of CPL asymmetry factors constructed by OIHPs single crystals.

**Figure 4:**
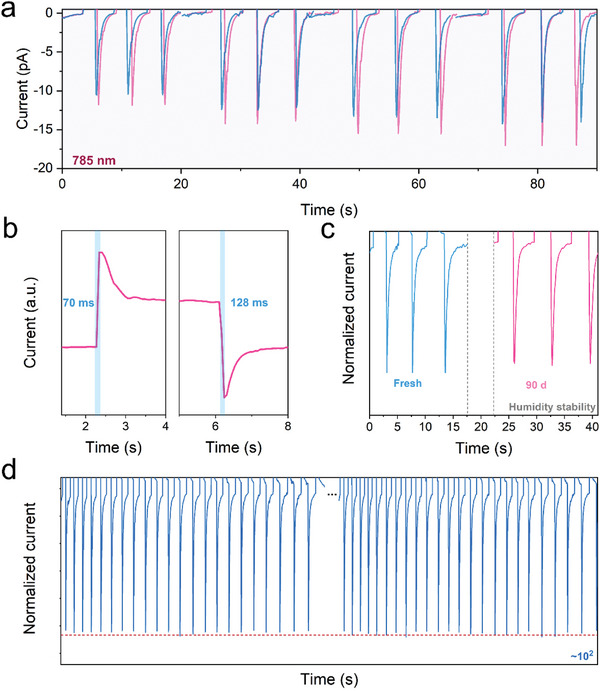
The photoexcited pyroelectric detection under 785 nm a) The photoexcited pyroelectric CPL detection under 785 nm with increased light power of 140, 180, 220, and 260 mW cm^−2^. b) The response time of **1‐S** is under 785 nm. c) The environmental stability of **1‐S** under 785 nm. d) The circle stability of **1‐S** under 785 nm.

Recently, the phenomenon of photo‐excited pyroelectricity, directly converting light‐induced temperature fluctuation into an electrical signal has attracted attention in the fields of sensing, energy harvesting, and optoelectronic.^[^ ^28^ ^]^ Thus, based on the pyroelectric semiconductor **1‐S**, self‐driven photopyroelectric detection was further performed along the polar axis (Figure S5, Supporting Information). As shown in Figure 2b,c, when the 405 nm laser is chosen as the heat source, **1‐S** exhibits a strikingly photoexcited pyroelectric behavior. Specifically, under 405 nm light irradiation, a prompt and pronounced transient photopyroelectric current rapidly emerged with a positive current value, while a distinct current with a negative value was detected at the moment the light was turned off. This photo‐excited pyroelectric behavior is mainly caused by spontaneous polarization changes induced by temperature fluctuations under on/off light.^[^ ^29^ ^]^ As shown in Figure 2c, under dark conditions, characterized by essentially constant temperature, the spontaneous polarization within **1‐S** along the polar axis can generate opposite bound charges on both ends and the free charges are in the electrodes to guarantee electric neutrality (dT/dt = 0, the I stage).^[^ ^30^ ^]^ As the 405 nm laser is turned on, two phenomena occur simultaneously, leading to a sharp current peak *I*_pyro+photo_. On the one hand, the light‐activated electron and hole pairs are generated and separated by the built‐in electric field induced by the polar structure, generating a photocurrent (*I*_photo_). On the other hand, the surface temperature of **1‐S** suddenly enhances under open‐light illumination due to the photothermal effect,^[^ ^31^ ^]^ which disturbed the random oscillation state of electric dipoles, leading to the decreased spontaneous polarization and fewer bound charges (dT/dt > 0; the II stage).^[^ ^32^ ^]^ As a result, the free charge at the interfaces is no longer completely shielded from the bound charges, which flow to the counter electrode and generate the positive output photopyroelectric current (*I*_pyro_). With continuous irradiation, the temperature gradually remains constant in **1‐S**, and the *I*_pyro_ progressively disappears, leaving only a stable *I*_photo_ (dT/dt = 0; the III stage). When turned off the laser, the temperature of **1‐S** suddenly drops, leading to an increase in spontaneous polarization, more bound charges, and the generation of a reverse photopyroelectric current (*I*_pyro’_) (dT/dt < 0; the IV stage).^[^ ^33^ ^]^ Significantly, photopyroelectric (*I*_pyro’_) only depends on the change of the electric dipole in response to photo‐induced temperature fluctuations and is independent of the bandgap absorption, which can break the limitations of material absorption for broad‐spectrum photoelectric detection (Figure 2d).^[^ ^27^ ^]^ Therefore, to explore photo‐excited pyroelectricity and ignore the influence of material absorption on photoelectric properties (*I*_photo_), our work mainly focuses on its photopyroelectric current (*I*_pyro’_) at the time of light‐off to research its photopyroelectric detection performance. As shown in Figure 2e, at the same optical power of 184 mW cm^−2^, **1‐S** shows a broadband pyro‐phototronic response, independent of material absorption, with significant *I*_pyro’_ in the 405–1550 nm range (The complete *I–t* curve is shown in Figure S6, Supporting Information). Probably due to the relatively small changes in spontaneous polarization caused by long‐wavelength irradiation, the photopyroelectric current (*I*_pyro’_) depends on the wavelength and decreases with the increasing incident wavelength.^[^ ^26^ ^]^ To the best of our knowledge, this is the longest light detection range achieved by 2D chiral hybrid perovskites. More importantly, the unique formamidine‐based multilayered **1‐S** with broadband spin‐selective absorption and transitions can combine the merits of intrinsic pyro‐phototronic effect for photopyroelectric‐based CPL detection without bias support (**Figure** **3**a).^[^ ^8^ ^]^ As depicted in Figures 2d and S7 (Supporting Information), the UV–vis absorption spectrum and spectral response measurements reveal the absorption edge of **1‐S** is ≈844 nm, making it one of the rare chiral materials with near‐infrared spin‐selected absorption. Thus, the photopyroelectric‐induced broadband CPL detection from 405 to 785 nm was performed on **1‐S**. Specifically, by adjusting the angle between a linear polarizer (λ/2) and a quarter‐wave (λ/4) plate, the left‐handed and right‐handed CPL was generated and then irradiated onto the **1‐S** detector (Figure 3a).^[^ ^34^ ^]^ As shown in Figures 3b–e and S8 (Supporting Information), **1‐S** shows broadband photopyroelectric‐based CPL detection performance under 405, 520, 637, and 785 nm with the light power of 65.5, 92.3, 85.2, and 120 mW cm^−^^2^, respectively (The different optical powers result from the different attenuation of incident light in different wavelengths after passing through a λ/2 and a λ/4 plate.). To analyze the CPL detection ability of **1‐S**, the anisotropic factor *g*_lph_ is calculated by the equation of *g*_lph_ = 2(*I*_L_ − *I*_R_)/(*I*_L_ + *I*_R_),^[^ ^35^ ^]^ where *I*_L_ and *I*_R_ are photopyroelectric currents (*I*_pyro’_) under LCP and RCP light. Under 405, 520, 637, and 785 nm the *g*_lph_ are respectively 0.24, 0.29, 0.26, and 0.14. The maximum *g*_lph_ of 0.29 under 520 nm is comparable with the reported efficient CPL detectors, such as (R)‐β‐ MPA]_2_MAPb_2_I_7_ (0.2 at 430 nm),^[^ ^11b^ ^]^ (S‐MBA)_2_PbI_4_ (0.24 at 510 nm),^[^ ^7a^ ^]^ (*R*/*S*‐3AMP)PbBr_4_ (0.3 at 520 nm)^[^ ^3a^ ^]^ (Figure 3f; Table S2, Supporting Information). According to the excellent photopyroelectric‐based CPL detection performance of **1‐S**, the related reasons are analyzed as follows. First, the intrinsic chiroptical activity of chiral perovskites facilitates the selective absorption of CPL. The preference for absorbing one type of CPL over the other is a key factor in differentiating between LCP and RCP.^[^ ^36^ ^]^ Second, upon absorption of CPL, chiral hybrid perovskites can efficiently convert the absorbed light energy into heat due to their photothermal properties.^[^ ^31^ ^]^ The selective CPL absorption induced by chiral hybrid perovskites’ chiroptical activity can result in different heat generation and consequently, different *I*_pyro’_ under LCP and RCP.^[^ ^37^ ^]^ Therefore, the photopyroelectric‐induced CPL detection capability of chiral hybrid perovskites is fundamentally linked to their chiral polar structure, which combines the merits of chiroptical activity and the pyro‐phototronic effect to detect and differentiate CPL's handedness.^[^ ^8^ ^]^ Emphatically, leveraging the broadband spin‐selective absorption of formamidine‐based **1‐S** up to NIR, this study pioneers the realization of photopyroelectric‐based NIR CPL detection in chiral OIHPs. Subsequently, the NIR photopyroelectric properties are deeply explored. As illustrated in **Figures** **4**a and S9 (Supporting Information), **1‐S** shows significant CPL photopyroelectric response (*I*_pyro’_) under 785 nm, which increases with the incident power, owing to intensified temperature fluctuations at higher optical power with the maximum *g*_lph_ of 0.20 under 260 mW cm^−2^.^[^ ^32^ ^]^ To explore the detector's versatility and reliability, the variations of CPL pyroelectric signals (*I*_pyro’_) at different temperatures are performed under 785 nm light illumination (power density of 220 mW cm^−2^) (Figure S10, Supporting Information). As the ambient temperature increases from 22 to 44 °C, the temperature gradient of photopyroelectric detectors decreases when the light is switched on and off, accompanied by the decreased pyroelectric current (*I*_pyro’_).^[^ ^38^ ^]^ Interestingly, under a high ambient temperature of 44 °C, the instrument still has CPL detection performance with a *g*_lph_ of 0.16, identifying its potential to work in different temperatures. Moreover, the response time, as an indispensable property for photodetectors, was measured under 785 nm illumination. As shown in Figure 4b, the decay time of on and off for **1‐S** is fast to 70 and 128 ms, surpassing those of reported 2D photodetectors.^[^ ^8^, ^26^ ^]^ Subsequently, the stability of the photopyroelectric device was investigated.^[^ ^28^ ^]^ As depicted in Figures 4c and S11 (Supporting Information), **1‐S** exhibited remarkable environmental stability under standard atmospheric conditions, with only a 7% degradation in *I*_pyro’_ after 90 days. Additionally, the *I*_pyro’_ maintains stability after ≈10^2^ cycles without any significant degradation (Figure 4d; Figure S12, Supporting Information), which shows its potential for future photopyroelectric‐based detectors in photoelectric and sensing fields. Importantly, the photopyroelectric performance can be modified by different conditions, including voltage, light intensity, and frequency. First, the variations of photopyroelectric signals under 785 nm light illumination (with a power density of 220 mW cm^−^^2^) at different bias voltages are shown in Figure S13 (Supporting Information). During the test, the positive and negative bias voltages were respectively applied. At 0 V bias, minimal dark current results in negligible Joule heat generation.^[^ ^39^ ^]^ Therefore, when switching the light on and off, the temperature gradient is maximum, resulting in significant pyroelectric signals. Applying a negative bias (−0.2 V) counteracts the built‐in electric field along the polar axis, inhibiting the separation of photogenerated carriers, and causing the photocurrent to nearly vanish during illumination.^[^ ^40^ ^]^ When the bias voltage decreases further to −1 V, carrier transport behavior becomes primarily influenced by this voltage. The photocurrent exhibits a sharp increase to −40 pA, while the pyroelectric signal becomes nearly undetectable due to significant thermal effects from the high current, disrupting the light‐induced temperature gradient in **1‐S**. When a positive bias voltage is performed from 0.2 to 30 V, the positive bias voltage promotes the effects of built‐in electric fields and enhances the separation of photogenerated carriers with intensified photocurrent. However, compared to the photocurrent, the pyroelectric signal decreases because the thermal effect of the current weakens the light‐induced temperature gradient in **1‐S**.^[^ ^39^ ^]^ Second, the variations of photopyroelectric signals at different light intensities under 785 nm light illumination are shown in Figure S14 (Supporting Information). Under zero bias, the photopyroelectric response (*I*_pyro’_) of **1‐S** gradually increases with the enhanced incident power, owing to intensified temperature fluctuations at higher optical power.^[^ ^28^ ^]^ Based on the pyro‐phototronic effect under different light intensities in Figure S14 (Supporting Information), the responsivity, and detectivity are respectively calculated with the maximum value of 0.51 µA W^−1^ and 1.33 × 10^8^ Jones, which is comparable with the reported photopyroelectric detectors^[^ ^8^, ^41^ ^]^ (Figure S15, Supporting Information). Then, the variations in pyroelectric signals at different frequencies under 785 nm light illumination are shown in Figure S16 (Supporting Information). With constant light intensity, the temperature difference should remain constant when switching. However, when enhancing frequency, the short and intense light interactions can lead to higher temperature gradients because energy is absorbed and converted more rapidly, thus strengthening the pyroelectric signal. As shown in Figure S16 (Supporting Information), the pyro‐phototronic signal increases as the frequency increases, reaching its maximum at 12 Hz. Due to the response time limitations of the photodetector, it cannot increase the temperature gradient in light beyond 12 Hz, resulting in a reduction of the signal at higher frequencies.^[^ ^39^ ^]^ Consequently, **1‐S** presents modified NIR photopyroelectric performance under different conditions, showing its excellent applicating potential in photopyroelectric detection fields.

## 3 Conclusion

In conclusion, this study, for the first time, realizes visible to near‐infrared photopyroelectric‐typed broadband CPL detection in chiral OIHPs. Specifically, the formamidine‐based chiral‐polar multilayered **1‐S** with intrinsic chiroptical activity and pyro‐phototronic effect showcases its potential for photopyroelectric‐based CPL detection. Notably, **1‐S** demonstrates outstanding self‐driven photopyroelectric‐based CPL detection performance, characterized by anisotropic factors (*g*_lph_) of 0.24, 0.29, 0.26, and 0.20 at wavelengths of 405, 520, 637, and 785 nm, respectively. Furthermore, the excellent environmental and response stability of **1‐S** under 785 nm illumination underscores its promise for practical applications. These findings not only advance the understanding of photopyroelectric‐based CPL detection mechanisms but also pave a new route for the development of innovative photonic and sensing technologies.

## Conflict of Interest

The authors declare no conflict of interest.

## Supporting information

Supporting Information

## Data Availability

The data that support the findings of this study are available from the corresponding author upon reasonable request.
