# Supplementary material for: Pyro‐Phototronic Effect Induced Circularly Polarized Light Detection with a Broadband Response
Source: Adv Sci (Weinh). 2024 Jul 23;11(36):2404403. doi: 10.1002/advs.202404403 (PMC11423216; doi:10.1002/advs.202404403)
Supplement: Supplementary file 1 — Supporting Information [file ADVS-11-2404403-s001.docx]

Supporting Information

Pyro-phototronic Effect Induced Circularly Polarized Light Detection with a Broadband Response

*Qianwen Guan, Zeng-Kui Zhu, Huang Ye, Chengshu Zhang, Hang Li, Chengmin Ji, Xitao Liu, and Junhua Luo ^*^*

**Experimental Section**

**Materials**. (*S*)-(-)-1-4-Bromophenylethylamine (S-BPEA, C_8_H_10_NBr, 98%, Aladdin), lead(Ⅱ) acetate trihydrate Pb(Ac)_2_·3H_2_O (99.5%, Aladdin), hydroiodic acid (Hl, 55%-58%, SCR), formamidine acetate (FA·Ac, 99%, Aladdin). All chemicals were procured commercially and utilized without additional purification.

**Synthesis and crystal growth:** Stoichiometric amounts of S-BPEA (2 mmol), FA·Ac (1 mmol), and Pb(Ac)_2_·3H_2_O (2 mmol) were dissolved in a mixture of aqueous HI (55-58%, 10 mL). The molar ratio of the reactants was precisely controlled to ensure the formation of the desired perovskite phase. Then, the solution was heated to 60 °C under constant stirring until complete dissolution of all reactants. The clear solution was then subjected to a slow cooling process at a cooling rate of 1 °C per day from 40 to 10 °C. This controlled cooling process allowed for the nucleation and growth of high-quality single crystals of **1-S**. The resulting crystals exhibited a characteristic red-black color.

**Single crystal and** **powder X-ray diffraction:** Powder X-ray diffraction (PXRD) measurements on the powder sample were performed using a Rigaku MiniFlex 600 diffractometer equipped with a Cu Kα rotating anode. Data were collected at room temperature over an angular range of 5° - 40° with a scan speed of 1.25°/min. Bulk single-crystal XRD data for a **1-S** single crystal were collected on a Rigaku SmartLab X-ray diffractometer equipped with a rotating anode source (Cu Kα radiation). The rocking curves data were collected at room temperature using a scan speed of 0.4°/min to assess the crystal quality.

**Thermogravimetric analysis (TGA) measurement:** TGA measurement was performed on a Netzsch STA449F3 thermal analyzer with a temperature range of 400~1200 K and a heating rate of 15 ℃ min^-1^.

**The optical and photoelectric measurements:** The diffuse reflection spectra of 1-S were obtained using a PerkinElmer Lambda 950 UV-VIS-NIR spectrometer. The UV-VIS-NIR diffuse reflectance spectrum can be used for bandgap calculation based on the Tauc equation^[1-3]^:

$${(F(R_{\infty})h\nu)}^{1/n}= A(h\nu- E_{g})$$

, where *h* is Planck's constant, *ν* is the frequency of light, *A* is the proportional constant, *E*_g_ is the bandgap energy, *F*(*R_∞_*) named the Kubelka-Munk function is equal to (1 – 𝑅_∞_)^2^⁄2𝑅_∞_, n is respectively 2 and 1/2 for indirect and direct bandgap semiconductors. When (*F*(*R_∞_*)*hν*)^2^ is the vertical coordinates and *hν* is the horizontal coordinates, the *X*-axis intercept is the bandgap of the material (Figure S2). Current-voltage curve measurements were performed on a high-precision tester (Keithley 6517B). Pyroelectric coefficient (Pe) calculation: First, the curve of current versus temperature (I-T) was obtained by pyroelectric instrumentation. Then, the Pe is calculated based on the formula Pe = ∂(∫J dt)/∂T^[4,5]^, where J is the current density, t is time, and T is temperature.

**Morphology Characterization:** The AFM and SEM images of **1-S** were measured on a Bruker Dimension ICON atomic force microscope and a JEOL JSM6700-F field emission scanning electron microscope, respectively.

.
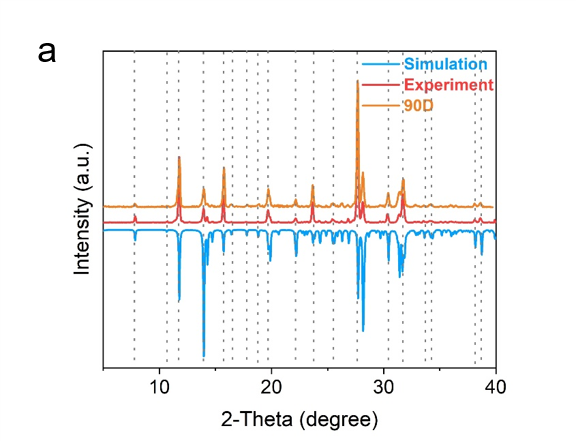

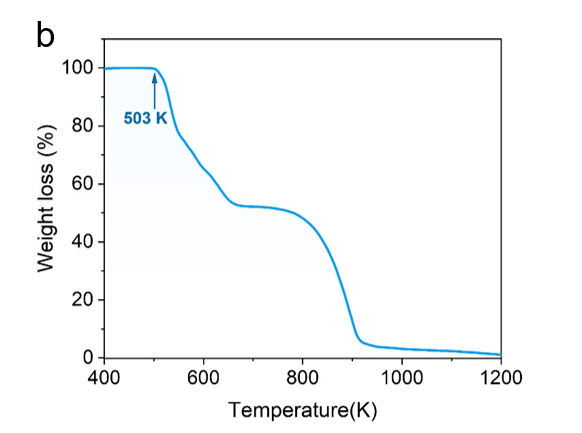


**Figure S1. (a).** The experimental (Fresh and 90 days’ crystal) and simulated powder X-ray diffraction image of 1-S. (b). The thermal gravity measurement of 1-S.

**
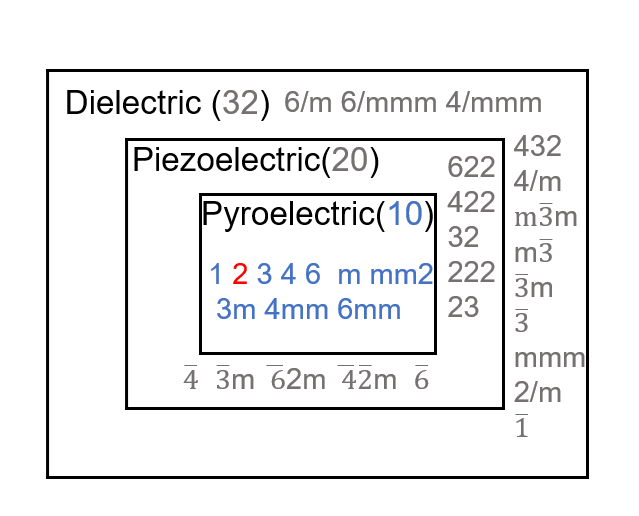
**

**Figure S2.** The point group of dielectric, piezoelectric, and pyroelectric materials.


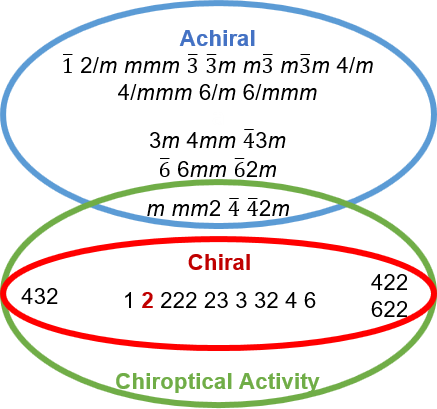


**
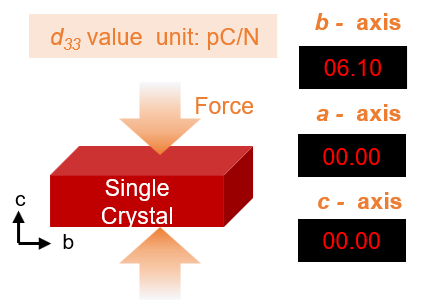
Figure S3**. The point group of materials with chirality and chiroptical activity.

**Figure S4**. The piezoelectric properties of **1-S** along different axes.


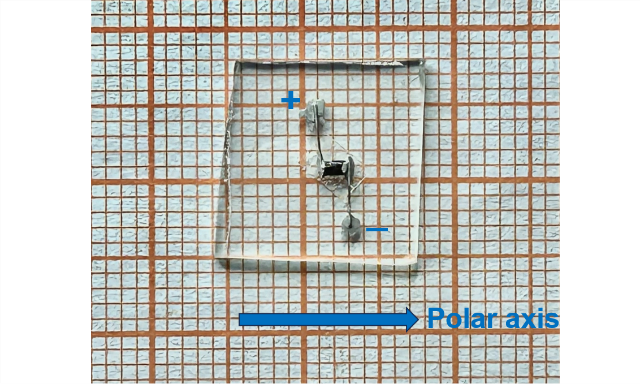


**Figure S5**. The electrode diagram of 1-S.


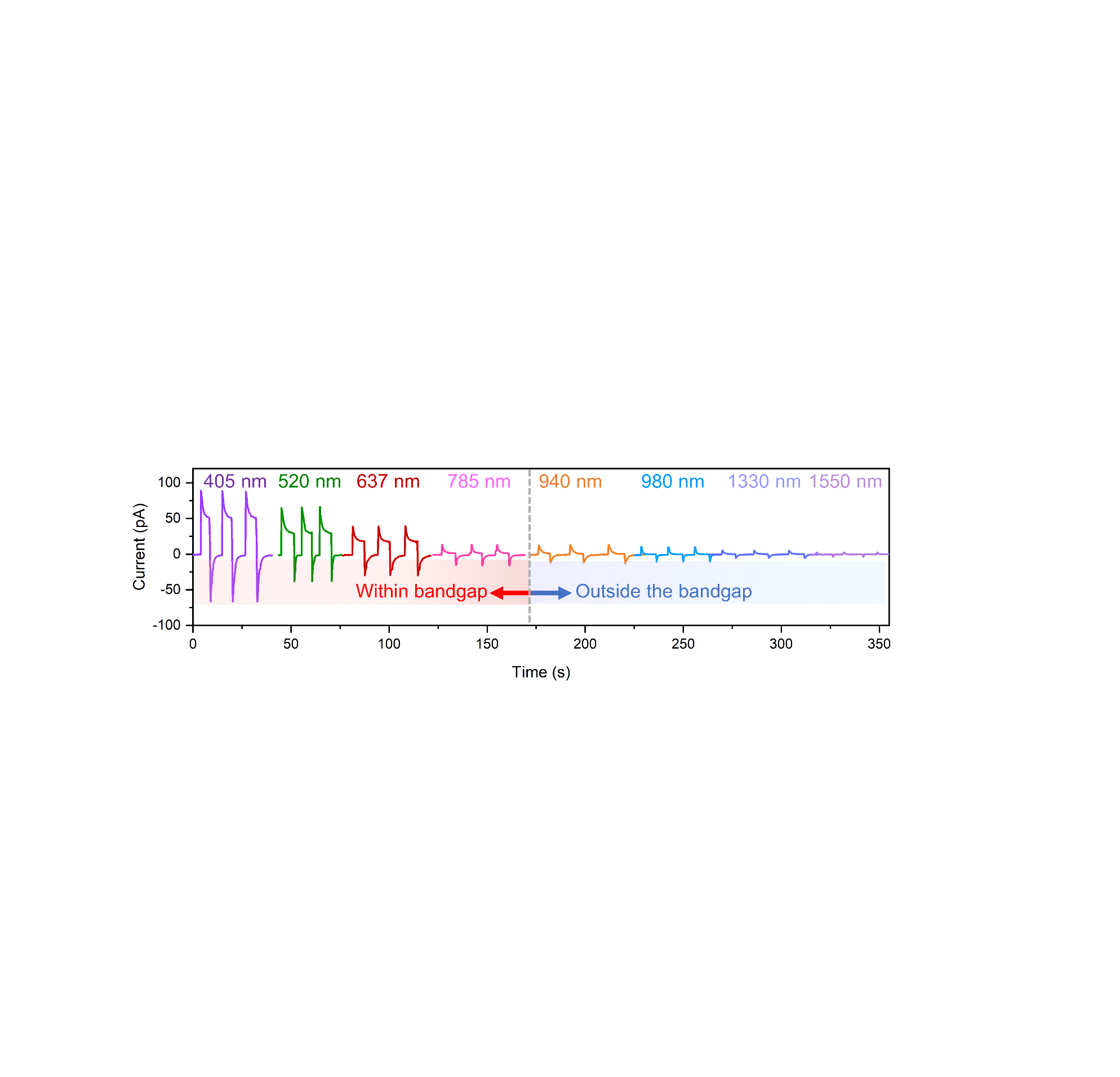


**Figure S6**. The whole I-t of Figure 2e.


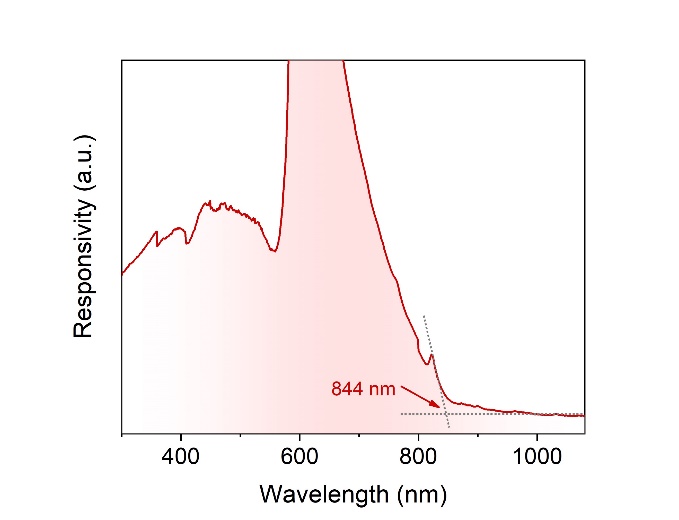


**Figure S7**. The spectral response of 1-S under 10 V bias.


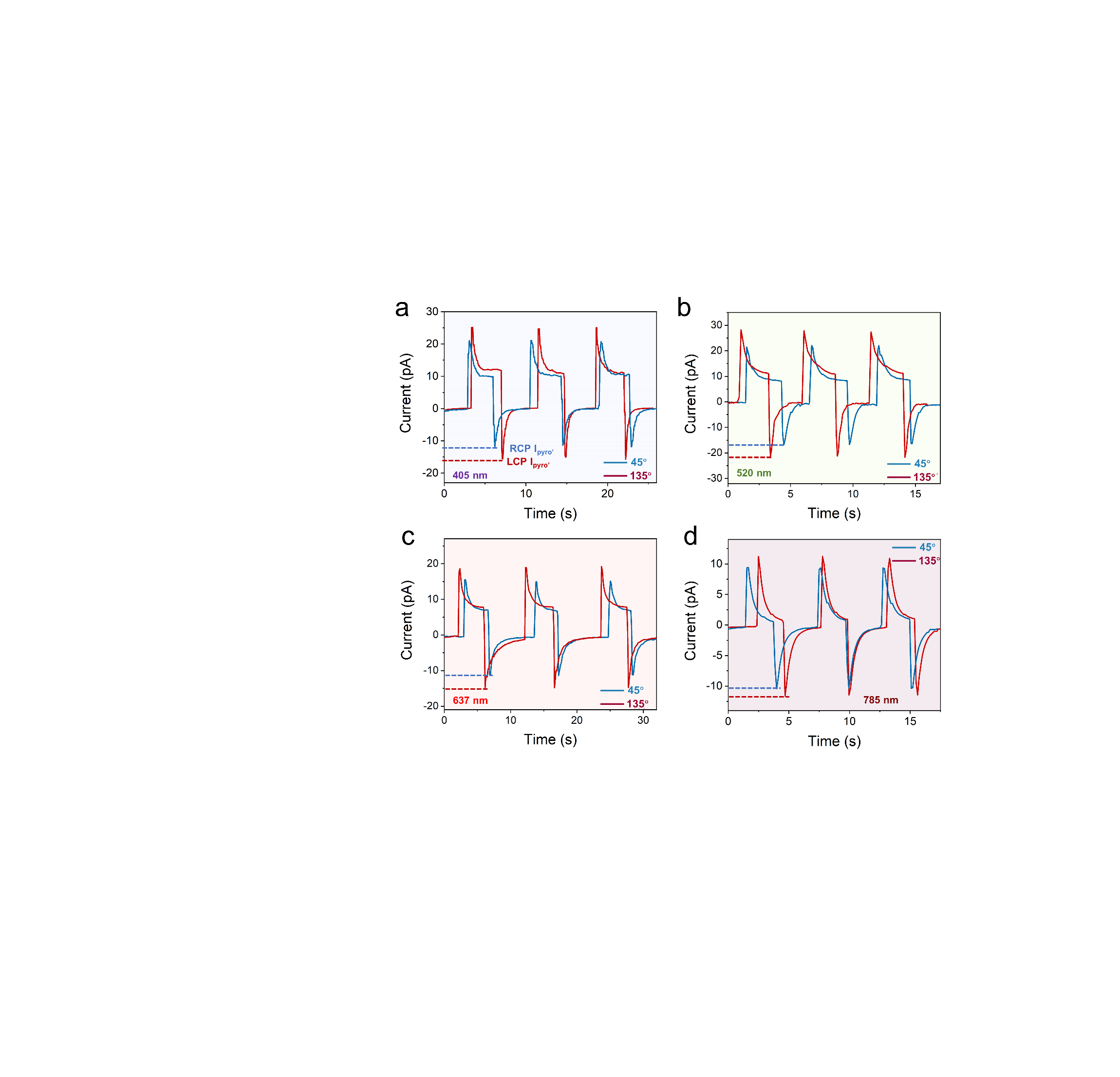


**Figure S8**. The whole I-t of (a). Figure 3b, (b). Figure 3c, (c). Figure 3d, and (d). Figure 3e.


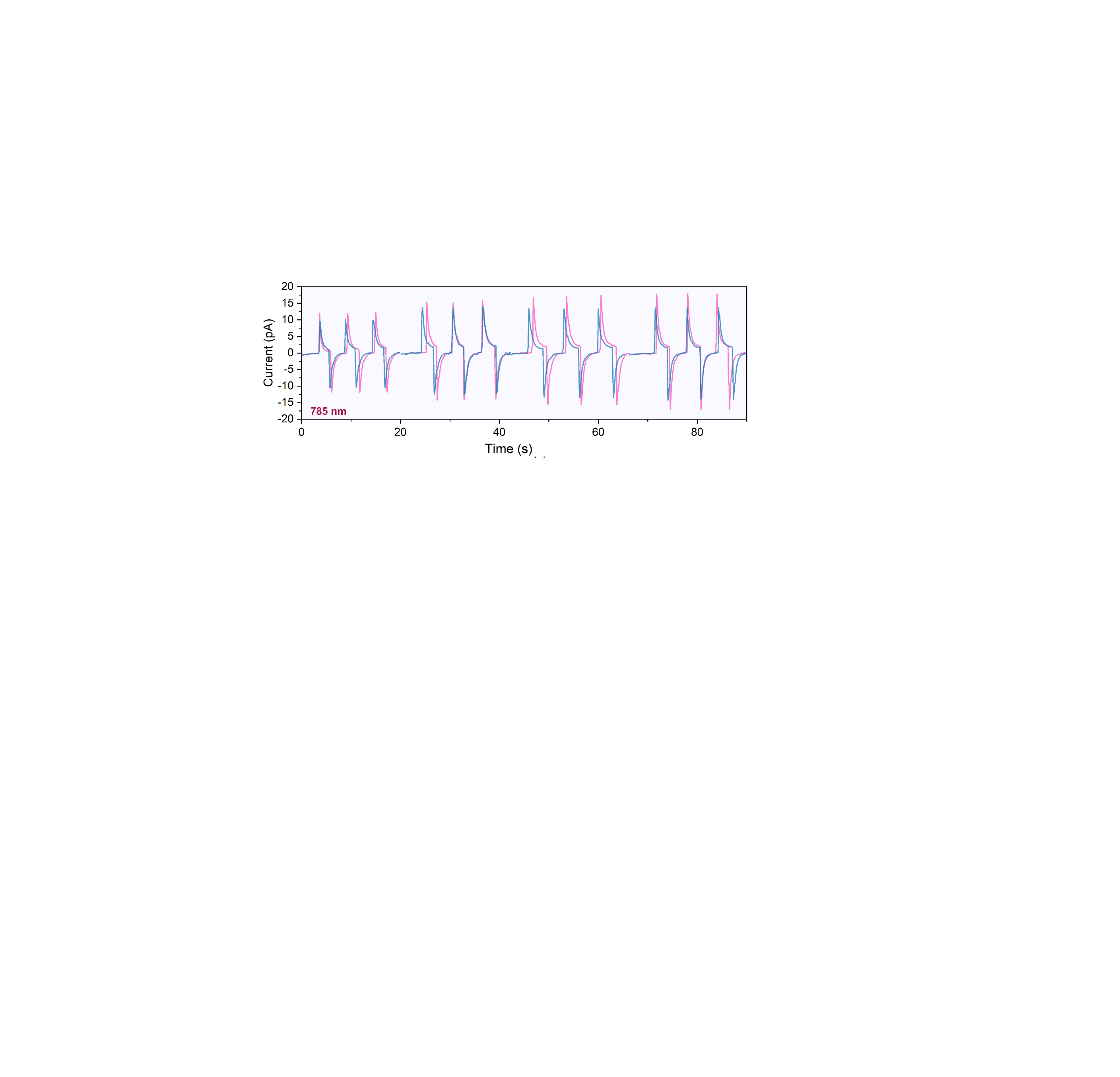


**Figure S9**. The whole I-t of Figure 4a.


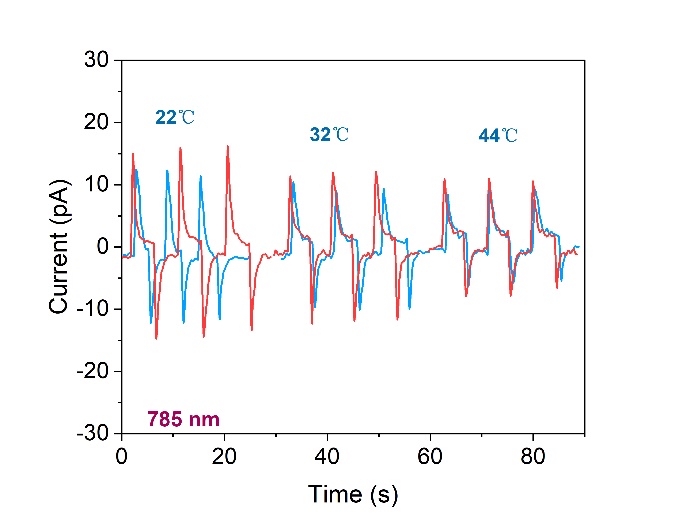


**Figure S10**. The photoresponse of the pyro-phototronic effect under different temperatures under 785 nm.


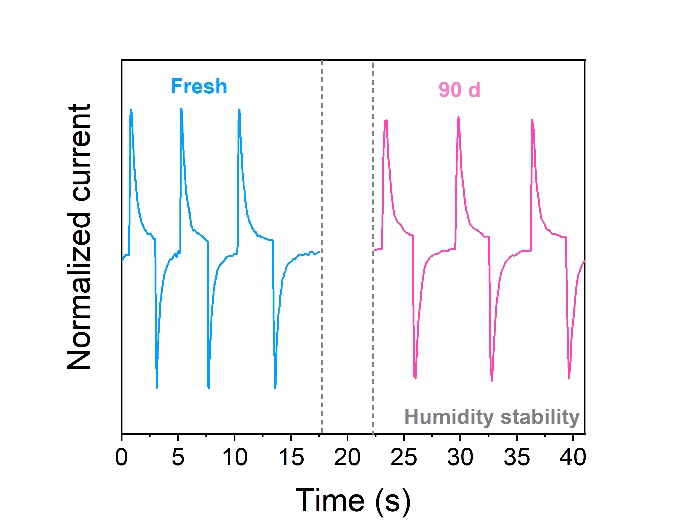


**Figure S11**. The whole I-t of Figure 4c.


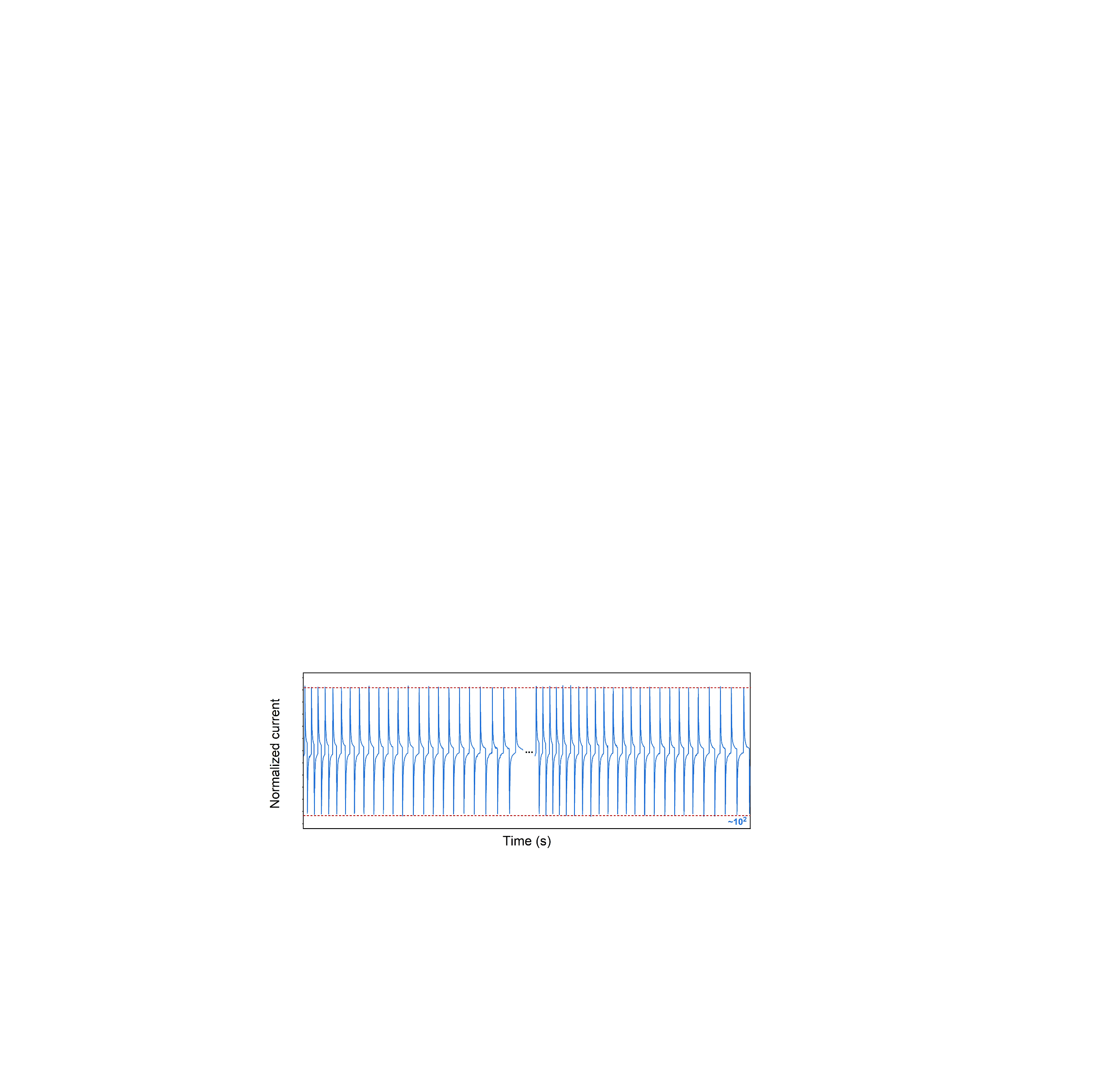


**Figure S12**. The whole I-t of Figure 4d.


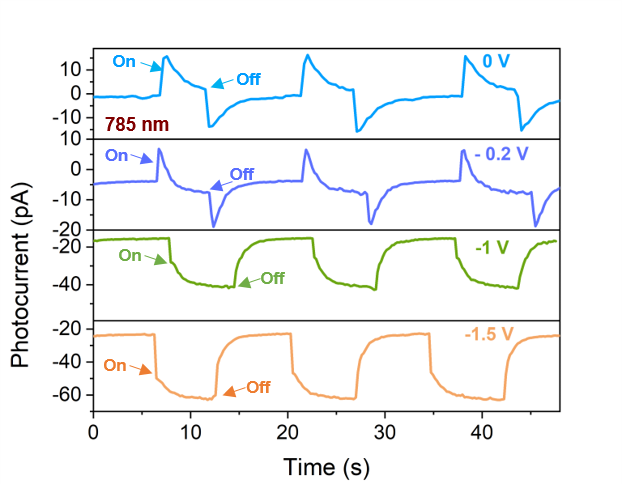

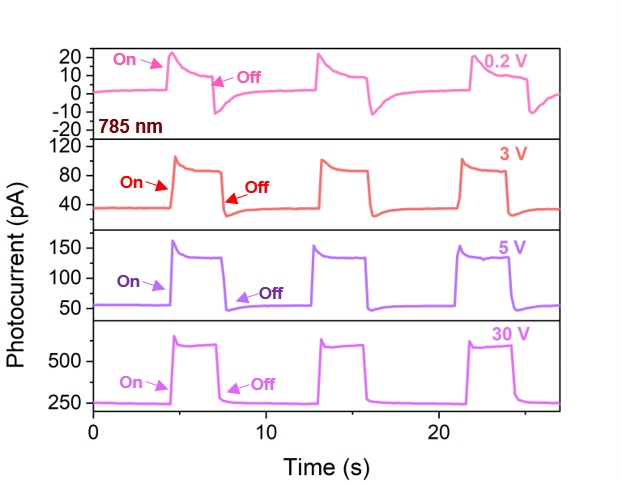


**Figure S13**. The photoresponse of the pyro-phototronic effect under negative and positive voltage under 785 nm.


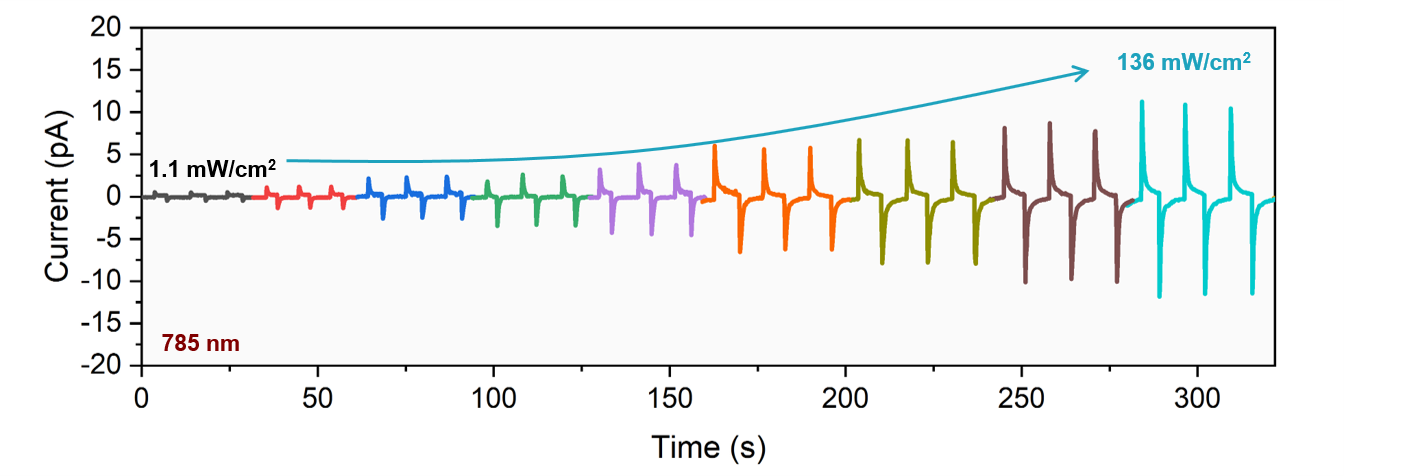


**Figure S14**. The photoresponse of the pyro-phototronic effect under different light intensities under 785 nm.


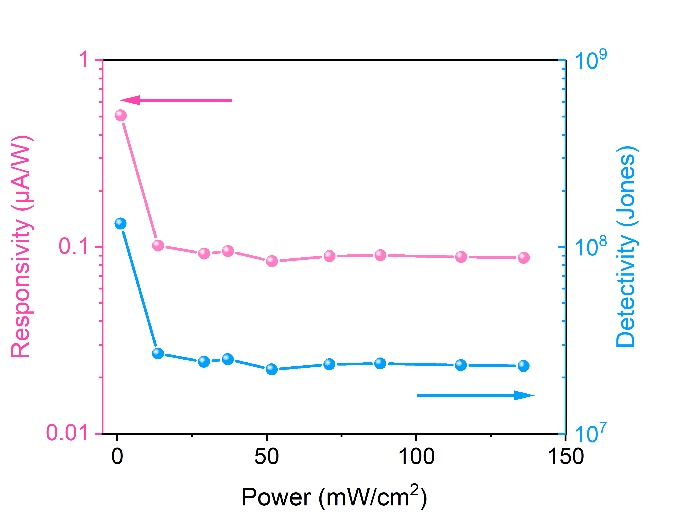


**Figure S15**. The responsivity and detectivity of 1-S under 785 nm.


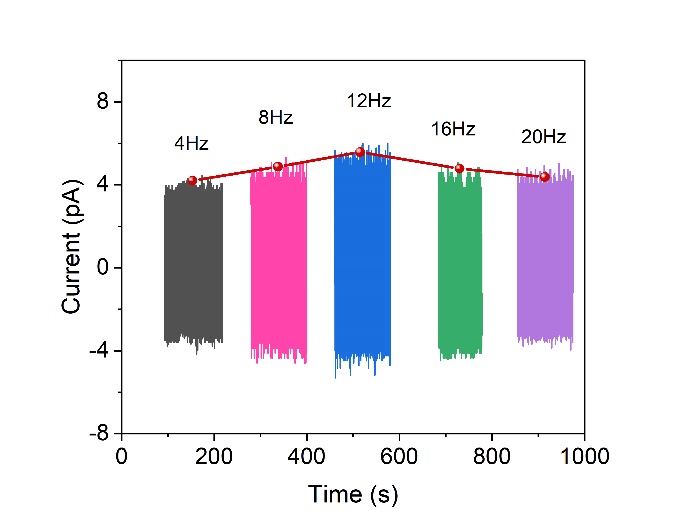


**Figure S16**. The photoresponse of the pyro-phototronic effect under different frequencies under 785 nm.

**Table S1.** **Crystal data and structure refinement of 1-S (CCDC number: 2193331)**

| Compound | **1-S** |
| --- | --- |
| Empirical formula | C_17_H_27_Br_2_I_7_N_4_Pb_2_ |
| Formula weight | 1749.92 |
| Temperature (*K*) | 250.05 |
| Space group | *P*2_1_ |
| Cell parameters | ***a*** = 9.0287(4) Å  ***b*** = 8.9165(4) Å  ***c*** = 22.5826(11) Å  ***α =*** 90°  ***β =*** 94.583(2)°  ***γ =*** 90° |
| *V* (Å^3^) | 1812.19(14) |
| *Z*, *ρ*_cal_. (g/cm^3^) | 2, 3.207 |
| *F*(000) | 1524 |
| Radiation λ (Mo/Ga Kα) (Å) | 0.71073 |
| Theta range (°) | 4.738 to 55.028 |
| Limiting indices | -11 ≤ h ≤ 11  -11 ≤ k ≤ 11  -28 ≤ l ≤ 29 |
| Reflections collected /unique | 29232 / 8293 [R(int) = 0.0579] |
| Data/restraints/parameter | 8293 / 22 / 270 |
| Final *R* indices [*I* > 2*σ*(*I*)]^a^ | *R*_1_ = 0.0376, *wR*_2_ = 0.1062 |
| *R* indices (all data) | *R*_1_ = 0.0504, *wR*_2_ = 0.1207 |

^a^R_1_ = Σ||F_o_| – |F_c_||/Σ|F_o_|, wR_2_ = [Σw(F_o_^2^ – F_c_^2^)^2^/Σ(F_o_^2^)^2^]^1/2^

**Table S2. The asymmetry factors of reported CPL detectors**

| **Materials** | **Dimension** | **Anisotropy factor**  **(g_Iph_)@wavelength** | **Power** | **Ref.** |
| --- | --- | --- | --- | --- |
| [(*R*)-β-MPA]_4_AgBiI_8_ single crystal (SC) | 2D | 0.3@520nm | 0 V | ^[6]^ |
| (R-/S-PPA)EA_2_Pb_2_Br_7_ SC | 2D | 0.3@266nm | 0 V | ^[7]^ |
| (SPPA)_4_(IPA)_6_Ag_2_Bi_4_I_24_⋅2H_2_O SC | 2D | 0.21@520nm | 1 V | ^[8]^ |
| (*R*-PPA)EAPbCl_4_ SC | 2D | 0.4@266nm | 0 V | ^[9]^ |
| (R-PPA)EAPbBr_4_ SC | 2D | 0.42@266nm | 0 V | ^[10]^ |
| (*R*-*β*-MPA)EAPbBr_4_ SC | 2D | 0.19@405nm | 0 V | ^[11]^ |
| (*R*-BPEA)_2_PbI_4_ SC | 2D | 0.13@520nm | 10 V | ^[12]^ |
| (*R*/*S*-3AMP)PbBr_4_ SC | 2D | 0.20@430 nm | 10 V | ^[13]^ |
| (*R*/*S*-BPEA)EA_6_Pb_4_Cl_15_ SC | 3D | 0.28@320nm | 10 V | ^[14]^ |
| (4-AMP)BiI_5_ SC | 1D | 0.24@405nm | 0 V | ^[15]^ |
| (*S*-α-MBA)_2_PbI_4_ NW array | 2D | 0.24@510nm | 5 V | ^[16]^ |
| [(*R*)-β-MPA]_2_MAPb_2_I_7_ film | 2D | 0.2@532nm | 5 V | ^[17]^ |
| (NEA)_2_(MA)_n‑1_Pb_n_I_3n+1_ film | 2D | 0.15@405nm | 20 V | ^[18]^ |
| (R-α-PEA)_2_PbI_4_ Nanowires | 2D | 0.15@505nm | 5 V | ^[19]^ |
| MAPbBr_3_-*R* | 3D | 0.39@405nm | 5 V | ^[20]^ |
| (R-NEA)PbI_3_ flake | 1D | 0.294@405nm | 4 V | ^[21]^ |
| (*R*-C_5_H_14_N)PbI_3_ microwire | 1D | 0.23@405nm | 5 V | ^[22]^ |

**References**

[1] Y. Qin, F. F. Gao, S. Qian, T. M. Guo, Y. J. Gong, Z. G. Li, G. D. Su, Y. Gao, W. Li, C. Jiang, P. Lu, X. H. Bu, *ACS Nano* **2022**, *16*, 3221-3230.

[2] C. Ji, S. Wang, Y. Wang, H. Chen, L. Li, Z. Sun, Y. Sui, S. Wang, J. Luo, *Adv. Funct. Mater.* **2020**, *30*, 1905529.

[3] F. Ye, H. Lin, H. Wu, L. Zhu, Z. Huang, D. Ouyang, G. Niu, W. C. H. Choy, *Adv. Funct. Mater.* **2019**, *29*, 1806984.

[4] L. Hua, J. Wang, Y. Liu, W. Guo, Y. Ma, H. Xu, S. Han, J. Luo, Z. Sun, *Adv. Sci.* **2023**, *10*, 2301064.

[5] W. Guo, H. Xu, W. Weng, L. Tang, Y. Ma, Y. Liu, L. Hua, B. Wang, J. Luo, Z. Sun, *Angew. Chem. Int. Ed* **2022**, *61*, e202213477.

[6] Y. Zhao, M. Dong, J. Feng, J. Zhao, Y. Guo, Y. Fu, H. Gao, J. Yang, L. Jiang, Y. Wu, *Adv. Opt. Mater.* **2021**, *10*, 2102227.

[7] T. Zhu, K. Zhang, C. Ji, X. Zhang, H. Ye, Y. Zou, J. Luo, *Small* **2022**, *18*, 2203571.

[8] Z.-K. Zhu, T. Zhu, J. Wu, S. You, P. Yu, X. Liu, L. Li, C. Ji, J. Luo, *Adv. Funct. Mater.* **2023**, *33*, 2214660.

[9] T. Zhu, H. Wu, C. Ji, X. Zhang, Y. Peng, Y. Yao, H. Ye, W. Weng, W. Lin, J. Luo, *Adv. Opt. Mater.* **2022**, *10*, 2200146.

[10] T. Zhu, W. Weng, C. Ji, X. Zhang, H. Ye, Y. Yao, X. Li, J. Li, W. Lin, J. Luo, *J. Am. Chem. Soc.* **2022**, *144*, 18062 – 18068.

[11] W. Wu, L. Li, D. Li, Y. Yao, Z. Xu, X. Liu, M. Hong, J. Luo, *Adv. Opt. Mater.* **2022**, *10*, 2102678.

[12] Y. Peng, X. Liu, L. Li, Y. Yao, H. Ye, X. Shang, X. Chen, J. Luo, *J. Am. Chem. Soc.* **2021**, *143*, 14077 – 14082.

[13] C.-C. Fan, X.-B. Han, B.-D. Liang, C. Shi, L.-P. Miao, C.-Y. Chai, C.-D. Liu, Q. Ye, W. Zhang, *Adv. Mater.* **2022**, *34*, 204119.

[14] Q. Guan, T. Zhu, Z. K. Zhu, H. Ye, S. You, P. Xu, J. Wu, X. Niu, C. Zhang, X. Liu, J. Luo, *Angew. Chem. Int. Ed.* **2023**, *62*，e202307034.

[15] T. Zhu, J. Bie, C. Ji, X. Zhang, L. Li, X. Liu, X.-Y. Huang, W. Fa, S. Chen, J. Luo, *Nat. Commun.* **2022**, *13*, 7702.

[16] Z. Liu, C. Zhang, X. Liu, A. Ren, Z. Zhou, C. Qiao, Y. Guan, Y. Fan, F. Hu, Y. S. Zhao, *Adv. Sci.* **2021**, *8*, 2102065.

[17] L. Wang, Y. Xue, M. Cui, Y. Huang, H. Xu, C. Qin, J. Yang, H. Dai, M. Yuan, *Angew. Chem. Int. Ed* **2020**, *59*, 6442-6450.

[18] T. Liu, W. Shi, W. Tang, Z. Liu, B. C. Schroeder, O. Fenwick, M. J. Fuchter, *ACS Nano* **2022**, *16*, 2682−2689.

[19] Y. Zhao, Y. Qiu, J. Feng, J. Zhao, G. Chen, H. Gao, Y. Zhao, L. Jiang, Y. Wu, *J. Am. Chem. Soc.* **2021**, *143*, 8437−8445.

[20] G. Chen, X. Liu, J. An, S. Wang, X. Zhao, Z. Gu, C. Yuan, X. Xu, J. Bao, H.-S. Hu, J. Li, X. Wang, *Nat. Chem.* **2023**, *15*, 1581–1590.

[21] M. Li, F. Fang, X. Huang, G. Liu, Z. Lai, Z. Chen, J. Hong, Y. Chen, R.-j. Wei, G.-H. Ning, K. Leng, Y. Shi, B. Tian, *Chem. Mater.* **2022**, *34*, 2955−2962.

[22] Y. Zhao, X. Li, J. Feng, J. Zhao, Y. Guo, M. Yuan, G. Chen, H. Gao, L. Jiang, Y. Wu, *Giant* **2021**, *9*, 100086.
